# A re‐innervated *in vitro* skin model of non‐histaminergic itch and skin neurogenic inflammation: PAR2‐, TRPV1‐ and TRPA1‐agonist induced functionality

**DOI:** 10.1002/ski2.66

**Published:** 2021-09-30

**Authors:** N. Lebonvallet, J. W. Fluhr, C. Le Gall‐Ianotto, R. Leschiera, M. Talagas, A. Reux, A. Bataille, C. Brun, T. Oddos, J.‐P. Pennec, J.‐L. Carré, L. Misery

**Affiliations:** ^1^ Laboratoire Interactions Epithéliums Neurones Université de Bretagne Occidentale Brest France; ^2^ Department of Dermatology Charité Universitätsmedizin Berlin Germany; ^3^ Johnson & Johnson Santé Beauté France Val de Reuil France; ^4^ Optimisation des Régulations PHYsiologiques Université de Bretagne Occidentale Brest France

## Abstract

**Background:**

Skin, and epidermis, is innervated by sensory nerve fibres. Interactions between them and signal transduction are only partially elucidated in physiological/pathological conditions, especially in pruritus.

**Objectives:**

To study the mechanisms involved in pruritus *in vitro*, we developed a skin explant model re‐innervated by sensory neurons.

**Methods:**

This model is based on the co‐culture of human skin explants and sensory neurons from dorsal root ganglia of rats. Innervation and the expression of protease activated receptor 2 (PAR2), transient receptor potential vanilloid 1 (TRPV1) and transient receptor potential ankyrin one (TRPA1) was analysed by immunostaining. The response of the model to TRPV1, PAR2 and TRPA1 agonists was analysed by patch‐clamp, qPCR and enzyme‐linked immunosorbent assay.

**Results:**

After 5 days of re‐innervating nerve fibres was evidenced in the epidermis. Re‐innervation was correlated with decrease of epidermal thickness and the number of apoptotic cells in the tissue. The major actors of non‐histaminergic itch (PAR‐2, thymic stromal lymphopoietin [TSLP], TSLP‐R, TRPA1 and TRPV1) were expressed in neurons and/or epidermal cells of skin explants. After topical exposure of TRPV1‐(Capsaicin), TRPA1‐(Polygodial) and PAR2‐agonist (SLIGKV‐NH_2_) activation of reinnervating neurons could be shown in patch‐clamp analysis. The release of TSLP was increased with capsaicin or SLIGKV but decreased with polygodial. Release of CGRP was increased by capsaicin and polygodial but decreased with SLIGKV. Activation by SLIGKV showed a decrease of VEGF; polygodial induced an increase of TSLP, Tumour necrosis factor (TNF) and nerve growth factor and capsaicin lead to a decrease of sema3 and TNF expression.

**Conclusion:**

The present model is suitable for studying itch and neurogenic inflammation pathways *in vitro*. We observed that activation of TRPV1, TRPA1 and PAR‐2 leads to different response profiles in re‐innervated skin explants.

## 1 INTRODUCTION

Skin and more specifically epidermis, is innervated by sensory nerve fibres. Keratinocytes and fibroblasts are in close contact with sensory nerves. However, interactions between them and signal transduction are only partially elucidated.^1^, ^2^ Sensory neurons, via neuropeptides and growth factors, plays an important role in epidermal homoeostasis and keratinocytes survival, proliferation or differentiation.^3^, ^4^, ^5^, ^6^, ^7^ On the other hand, keratinocytes produce neurotrophic or modulative factors allowing the survival of neurons as well as the maintenance, branching or growth of terminal nerve fibres.^8^, ^9^, ^10^, ^11^ Very few models were proposed to study these interactions.^12^, ^13^, ^14^ However, none of them was able to serve simultaneous evaluation of both morphologic and functional aspects, for example histology, electrophysiology or cytokines release and signal transduction.

**Key points**

**What is already known about this topic?**

- Co‐culture between neurons are already existing with human skin explant and dorsal root ganglia cells, or with reconstructed skin and sensory neurons. However, all techniques of analyses are not realizable for all models, furthermore, reinnervation of epidermis by sensory neuron is lacking or very weak. Interactions between them and signal transduction are only partially elucidated in physiological/pathological conditions, especially in pruritus.

**What does this study add?**

- This study is the first model with high and measurable reinnervation in the epidermis. The model permit to combine multiple combined techniques including electrophysiology. The investigation show activation of distinct pathway of PAR, TRPV1 and TRPA1.

An approach towards a complete 3D‐model to study epidermis–neuron interactions was introduced. Herein, we present the morphologic and functional validation of a model of re‐innervation of human skin explants by sensory neurons allowing to study the intimate mechanisms of non‐histaminergic pruritus pathway and skin neurogenic inflammation, which are closely intricated.^15^, ^16^, ^17^, ^18^, ^19^, ^20^ This model may serve as an alternative to animal testing and as screening model for itch modulation prior to human studies.

The following parameters were implemented in the functionality of our model: protease activated receptor 2 (PAR2), a G‐coupled receptor activated by proteases (trypsin and some kallikreins). PAR2 is expressed at the cellular membrane by the majority of cells including keratinocytes and neurons.^21^ It is implicated in inflammation, and concerning pruritus it is known as the main actor of non‐histaminergic pruritus particularly in atopic dermatitis (AD).^15^, ^17^, ^22^ The synthetic peptide SLIGKV permits the specific activation of PAR2. This activation in keratinocyte induces the release of thymic stromal lymphopoietin (TSLP) and several additional cytokines. PAR2 was selected to validate the model as relevant for the non‐histaminergic pruritus pathway with TSLP as read out. TRPV1 (transient receptor potential vanilloid 1) is an ionotropic channel, with high affinity to calcium. It is expressed at the extracellular plasma membrane or intra‐plasmatic, for example in the endoplasmic reticulum in the majority of cells including keratinocytes and C‐fibres neurons.^23^, ^24^, ^25^ TRPV1 is considered as pain/itch receptor and is activated by heat (>43°C), protons or vanilloid derivatives. For example, capsaicin, a natural TRPV1 agonist is a component of chili pepper. transient receptor potential ankyrin one (TRPA1) is an ionotropic, channel, with high affinity to calcium. It is considered as pain/itch receptor and may be activated by cold (<17°C) and different natural compounds, for example menthol, AITC ‘Allyl isothiocyanate' or polygodial. TRPV1 and TRPA1 may be both activated or activate downstream effects directly or after the interaction with the G‐coupled receptor like PAR2.^16^, ^25^ TRPV1‐and TRPA1 activation in neurons induces the release of neuropeptides including SP (Substance P) and calcitonin‐gene related peptide (CGRP) as well as the production of pro‐inflammatory cytokines. TRPV1 and TRPA1 are main actors of neurogenic inflammation.^25^ They were selected to validate the model as neurogenic inflammation model, with CGRP as read‐out. In the present study, lactic acid (LA) was used as a non‐specific inflammation inducer. LA is used in stinging test to induce unpleasant sensations (e.g. pain, itch, stinging, pickling and burning) in patients with sensitive skin. It is known as a potent activator of ASIC (acidic sensitive ionic channel) and TRPV1. *In vitro*, the use of LA induces the release of SP in neurons and keratinocytes.^26^ Tumour necrosis factor alpha (TNF‐α) is a cytokine used as a general inflammation marker produced by majority of cells and inducing several pro‐inflammatory pathways. TSLP is a major cytokine involved in non‐histaminergic pruritus particularly in AD.^15^, ^22^ TSLP is produced by differentiated and proliferating keratinocytes under the stimulation of PAR2. Hence TSLP via TSLP receptor (TSLPR) expressed on skin sensory fibres promotes pruritic signalling. Nerve growth factor (NGF), is implicated in the survival of neurons and promotes the neural length elongation in the central and peripheral nervous system. Semaphorin3a, in contrast is involved in the diminution of neurite length. NGF is equally implicated in the proliferation of keratinocytes. In AD, hyperproliferation of keratinocytes and increased nerve fibres in the epidermis are modulated by NGF/SEMA3A homoeostasis.^9^, ^27^ We selected these markers to identify the modulation of neuronal trophic response in the context of inflammation.

VEGF (vascular endothelial growth factor), a mediator of angiogenesis and inflammation, is implicated in AD, psoriasis and neurogenic inflammation.^28^, ^29^ EGF (epidermal growth factor), is involved in the proliferation of keratinocytes and regulates inflammation.^30^, ^31^ MT5‐MMP (membrane type 5 – Matrix metalloprotease) is expressed by CGRP‐containing peptidergic nociceptors in dorsal root ganglia (DRG). The absence of MT5‐MMP enhances sensitivity to noxious thermal stimuli under basal conditions.^32^ BDNF ‘brain derived neurotrophic factor' is a protein implicated in survival of neuron and neurite length. Its implication in pain and itch is still under discussion.^8^, ^33^, ^34^ CGRP is one of main peptides involved in neurogenic inflammation. CGRP is released after TRPV1 or TRPA1 activation and stimulates the proliferation of keratinocytes.

In this article, we propose a morphological validation of the reinnervated skin model associated with an analysis of the implication of neurons in epidermal homoeostasis. Furthermore, we show that the model is electro‐physiologically functional and allows to monitor the modulation of cytokine release in the non‐histaminergic pruritus pathway induced by TRPV1‐, TRPA1‐and PAR2‐agonists. Equally, after induction of these receptors, we study the transcriptional response of sensory neurons or the skin. Selected trophic factors, cytokines, growth factors and neuropeptides implicated in inflammation, pruritus and skin homoeostasis were analysed.

## 2 RESULTS

### 2.1 Validation of re‐innervated human skin explant model with sensory neurons

**FIGURE 1:**
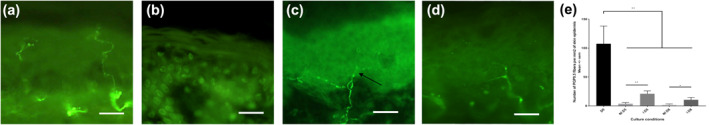
Visualization of re‐innervation of explanted skin by PGP9.5 immunostaining. Scale bars: 25 μm. (a) Skin explant with autologous sensory nerve endings in the epidermis right after explantation. (b) Non‐re‐innervated skin explant after 5 days of culture (explant cultured without sensory neurons): disappearance of nerve endings in the epidermis. (c) Skin explant cultured with sensory neurons after 5 days: reappearance of nerve endings, issued from dorsal root ganglia (DRG) cells. (d) Skin explant culture with sensory neurons at 8 days: new nerve endings are preserved. (e) Represents the number of PGP9.5+ nerve fibres per millimetre square of epidermis in sensory neurons from DRG (D0; *n* = 8), after 5 days of culture without sensory neurons (NID5; *n* = 8), with sensory neurons (ID5; *n* = 7); after 8 days of culture without sensory neurons (NID8; *n* = 6); with sensory neurons (ID8; *n* = 6). Values in (e) represent mean ± SEM. *p < 0.05. **p < 0.01

Our initial step was to re‐innervate a human skin explant with cultured sensory neurons from rat DRG. We have previously shown cultivation of sensory neurons isolated from rat DRG in co‐culture with human skin explants.^6^ The first part of our study was intended to validate the model on an extended descriptive level. Figure 1a visualizes sensory nerve fibres in the human skin explant by PGP9.5 immunostaining. The sensory nerve fibres were no longer detectable after 5 days (Figure 1b) of incubation without co‐culture of sensory neurons. In contrast, co‐culture with extracted sensory nerves lead to ingrowth after 5 days (Figure 1c) which was stable over the following 3 days (day 8; Figure 1d). Statistical analysis revealed a significant staining of sensory nerve fibres both at day 5 and day 8 in the co‐culture compared to non‐co‐cultured skin explants. However, the number of nerve fibres per millimetre square was significantly lower in the model than in the skin explant right after explantation (Figure 1e).

**FIGURE 2:**
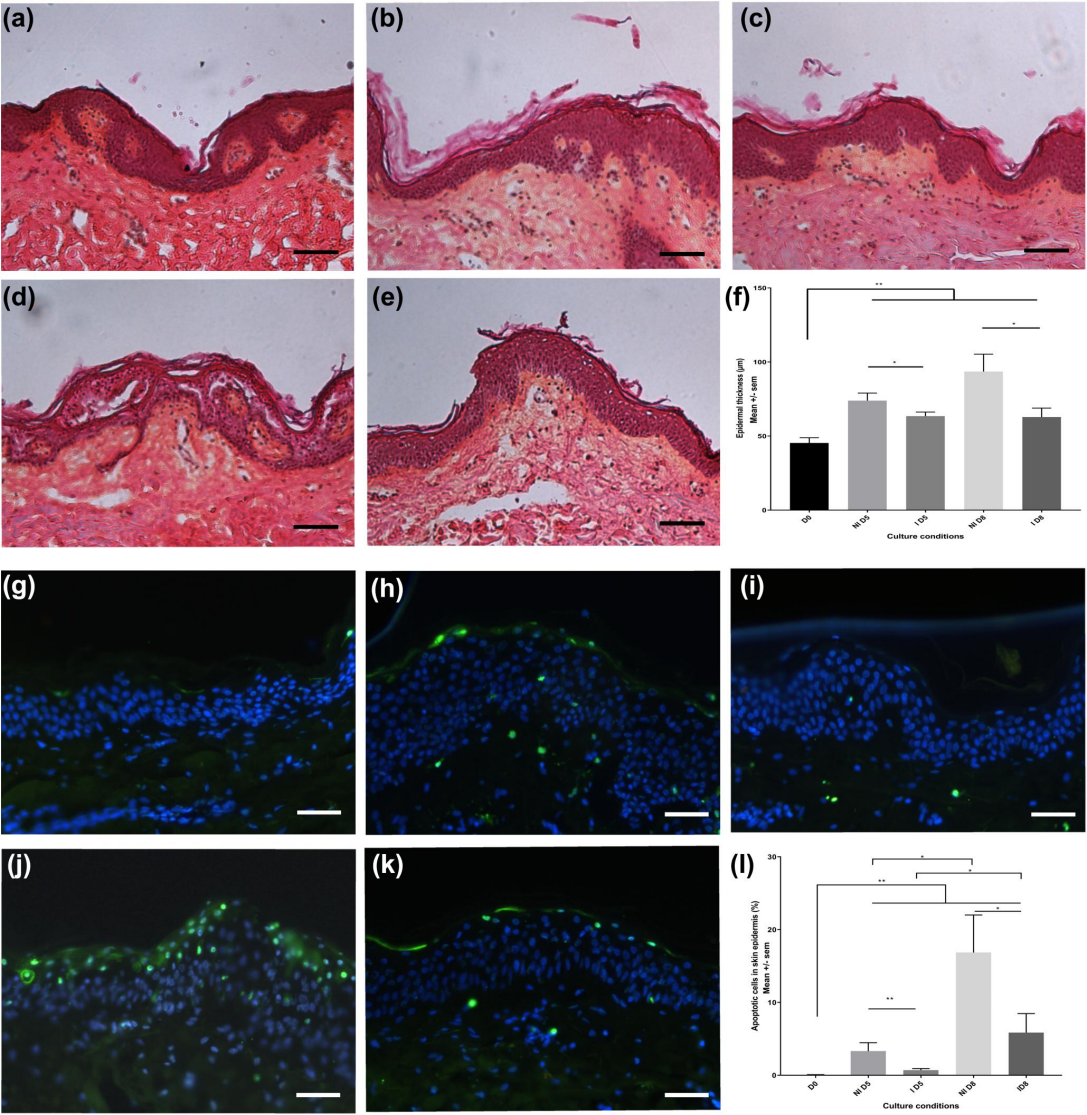
Epidermal thickness and apoptosis decreases after re‐innervation with sensory neurons. (a) Skin explant observed before culture. (b) Non‐reinnervated skin explant after 5 days of culture (explant cultured without sensory neurons). (c) Skin explant cultured with sensory neurons at 5 days. (d) Skin explant culture without sensory neurons at 8 days. (e) Skin explant culture with sensory neurons at 8 days. Scale bars: 50 μm. (f) Graphic represent the mean ± SEM of epidermal thickness in skin explant before culture (D0; *n* = 9), after 5 days of culture without (NID5; *n* = 9) or with sensory neurons (ID5; *n* = 9) and after 8 days of culture without (NID8; *n* = 8) or with sensory neurons (ID8; *n* = 8). Skin explants were stained with Haematoxylin‐Eosin. *p < 0.05. **p < 0.01. Analysis of epidermal apoptosis (TUNEL method, green; nuclei stained with DAPI, blue): (g) Skin explant observed before culture. (h) Non re‐innervated skin explant after 5 days of culture (explant cultured without sensory neurons). (i) Skin explant cultured with sensory neurons at 5 days. (j) Skin explant culture without sensory neurons at 8 days. (k) Skin explant culture with sensory neurons at 8 days. Scale bars: 50 μm. (l) Graphic represent the mean ± SEM of percentage of epidermal apoptotic cells in skin explant before culture (D0; *n* = 9), after 5 days of culture without (NID5; *n* = 9) or with sensory neurons (ID5; *n* = 9) and after 8 days of culture without (NID8; *n* = 8) or with sensory neurons (ID8; *n* = 8). *p < 0.05. **p < 0.01

The current study showed decrease in epidermal thickness at day 5 (Figure 2c,f) which was significantly lower than the non‐innervated skin explant (Figure 2b,f). The significant (*p* < 0.05) increase of epidermal thickness in the non‐innervated explants was partially normalized in the re‐innervated skin explants but still significantly higher (*p* < 0.05) (Figure 2f) than in the freshly excised skin samples (Figure 2a). The model showed an increase in apoptotic cells over time in all culture conditions compared to the freshly excised tissue (Figure 2). This increase could be mainly prevented in the early time point (Figure 2i,l) but with the increase of culture time apoptosis was further induced, however to a lesser degree in the re‐innervated skin explants (Figure 2k,l).

**FIGURE 3:**
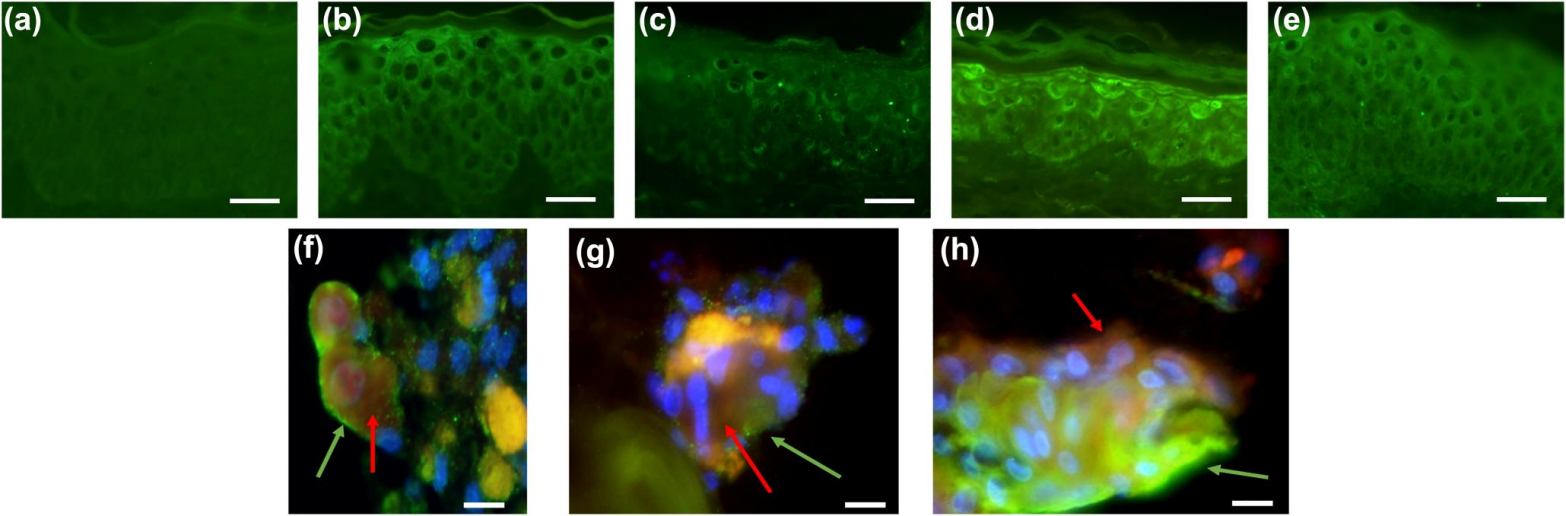
Expression of inflammation and non‐histaminergic pruritus marker on epidermis and clustered sensory neurons. Immunostaining with relevant markers involved in itch pathophysiology after 5 days of culture on re‐innervated skin explant (a) control without primary antibody, (b) protease activated receptor 2 (c) transient receptor potential ankyrin one (TRPA1) (d) transient receptor potential vanilloid 1 and (e) thymic stromal lymphopoietin (TSLP). Scale bars: 25 μm. On the bottom of the picture, immunostaining of sensory neurons organized in cluster under the skin explants and re‐innervating. Images represent the merged pictures of PGP9.5 (in red) immunostaining with green for (f) TSLPR; (g) TRPA1 and (h) IL31R (white arrows in the three figures). Nuclei appear in blue (DAPI). Scale bars: 5 µm

The expression of receptors or cytokines involved in pruriception was studied under the optimal co‐culture conditions after 5 days. The specific immunostaining of PAR2 (Figure 3b), TRPA1 (Figure 3c), TRPV1 (Figure 3d) and TSLP (Figure 3e) was positive for all of them. Subsequently we looked into spatial expression of TRPA1, TSLP and IL31 receptors (IL31R) at day 5 of co‐culture. We could observe a clustered expression of TSLPR (Figure 3f); TRPA1 (Figure 3g) and IL31R (Figure 3h) at the bottom of the contact zone of the sensory neurons and the dermis.

### 2.2 Electrophysiological functionality and biochemical response

**FIGURE 4:**
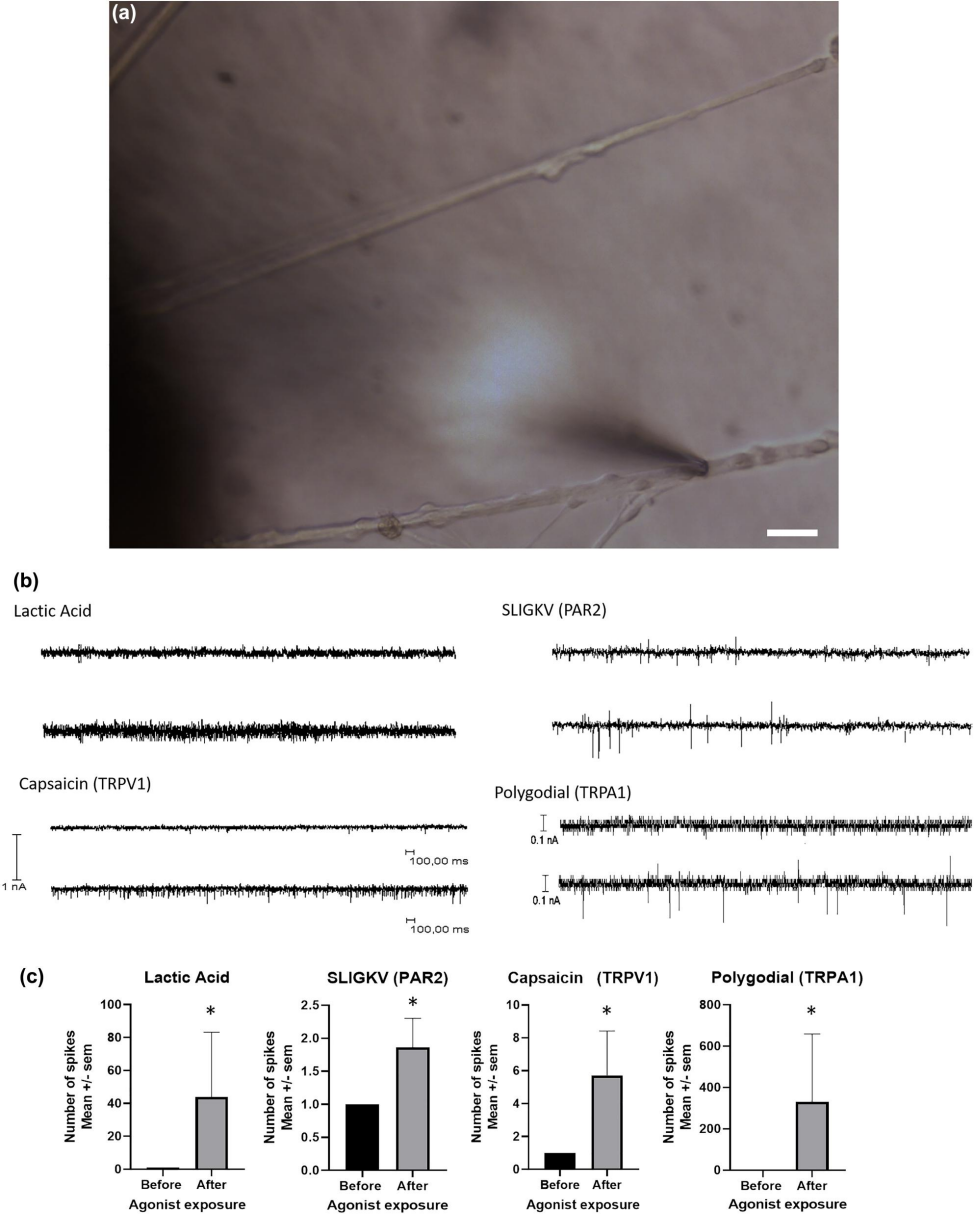
Electrophysiological response of sensory neurons. (a) Picture of patch‐clamp micropipette clamped on sensory neurons emitting an extension in direction of skin explant. Scale bar = 5 μm. (b) The innervation curves are representative records of nerve fibre activities with different agonists. For each used agonist, the upper trace corresponds to fibre activity before the exposure of skin explant to the different agonists. The bottom trace corresponds to the same record after the exposure to the agonist. (c) Number of spikes corresponding to electrical activity on the sensory neurons before and after exposure to agonists of responding fibres. The number of spikes compared to the control before agonist exposure with a pre‐selected value of 1. The values represent mean ± SEM

The electrophysiological functionality of the neurons was assessed in patch‐clamp experiments (Figure 4a). Representative patch‐clamp innervation curves before and after topical exposure of skin epidermis to specific and non‐specific agonists are depicted in Figure 4b. An increase in neuronal activity (spikes) was recorded for all agonists. Figure 4c represents the number of spikes (normalized to a value of 1 for non‐exposed neurons) before and after agonist exposure. The spike increase was significant for all compounds (*p* < 0.05).

**FIGURE 5:**
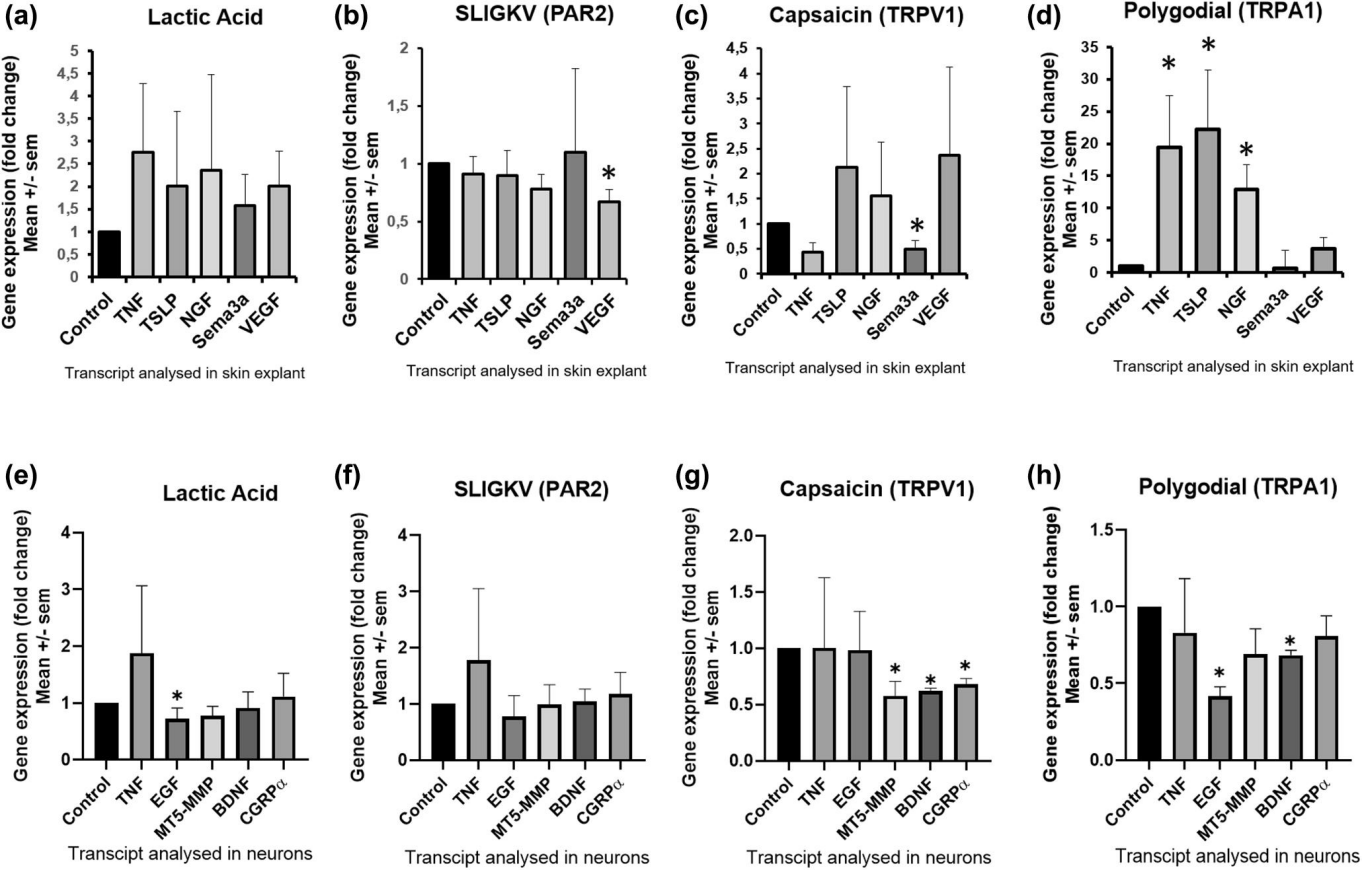
Gene expression modulation in skin explant or neurons after agonist exposure in re‐innervated skin explant model. Panel up: gene expression (of cytokines and trophic factors) on the transcriptional level (mRNA) as response of skin explant to an exposure to different receptor agonists at day 5 in the re‐innervated skin model. Solvents served as control condition and where pre‐set at a value of *x* = 1. (a) Gene expression after exposure to the unspecific activator lactic acid (10%) compared to culture medium. (b) Gene expression after SLIGKV (100 μM) exposure compared to phosphate‐buffered saline (PBS). (c) Gene expression after capsaicin (10 μM) exposure compared to ethanol. (d) Gene expression after polygodial (3 µM) compared to ethanol. *p < 0.05. *n* = 3 for each group; values present mean ± SEM. Panel down: Gene expression (of cytokines, trophic factors or neuropeptides) on the transcriptional level (mRNA) as response of sensory neurons to an exposure to different receptor agonists at day 5 in the re‐innervated skin model. Solvents served as control condition and where pre‐set at a value of *x* = 1. (e) Gene expression after exposure to the unspecific activator lactic acid (10%) compared to culture medium. (f) Gene expression after SLIGKV (100 μM) exposure compared to PBS. (g) Gene expression after capsaicin (10 μM) exposure compared to ethanol. (h) Gene expression after polygodial (3 µM) compared to ethanol. *p < 0.05. *n* = 3 for each group; values present mean ± SEM

Subsequently, we assessed the modulation of gene expression (qPCR) on the transcriptional level (mRNA) for cytokines, growth factors and mediators (Figure 5). A non‐specific agonist (LA) was used as initial compound. We studied two different areas of the re‐innervated skin explant at day 5: Figure 5a–d represents the upper epidermal area while Figures 5e–h shows data from the DRG‐seeding region. The values were normalized to the value of 1 for the corresponding solvent of the agonist. In the re‐innervated upper epidermal tissue, LA induced an increase in gene expression in all tested parameters without reaching the significance level (Figure 5a). SLIGKV (PAR2‐agonist), induced a significant decrease of VEGF gene expression (Figure 5b). For capsaicin (TRPV1‐agonist); a non‐significant increase was observed for almost all parameters except for TNF‐α (non‐significant decrease) and Sema3a (significant decrease) (Figure 5c). Polygodial (TRPA1‐agonist) induced a significant increase for TNF‐α, TSLP and NGF (Figure 5d).

In the DRG‐seeding region, LA induced a non‐significant increase in gene expression of TNF‐α and a significant decrease of EGF expression (Figure 5e). For SLIGKV no significant change in gene expression was observed (Figure 5f). For capsaicin significant decrease for MT5‐MMP, BDNF and CGRPα was recorded (Figure 5g). Polygodial induced a decrease for all parameters which reached the significance level for EGF and BDNF (Figure 5h).

**FIGURE 6:**
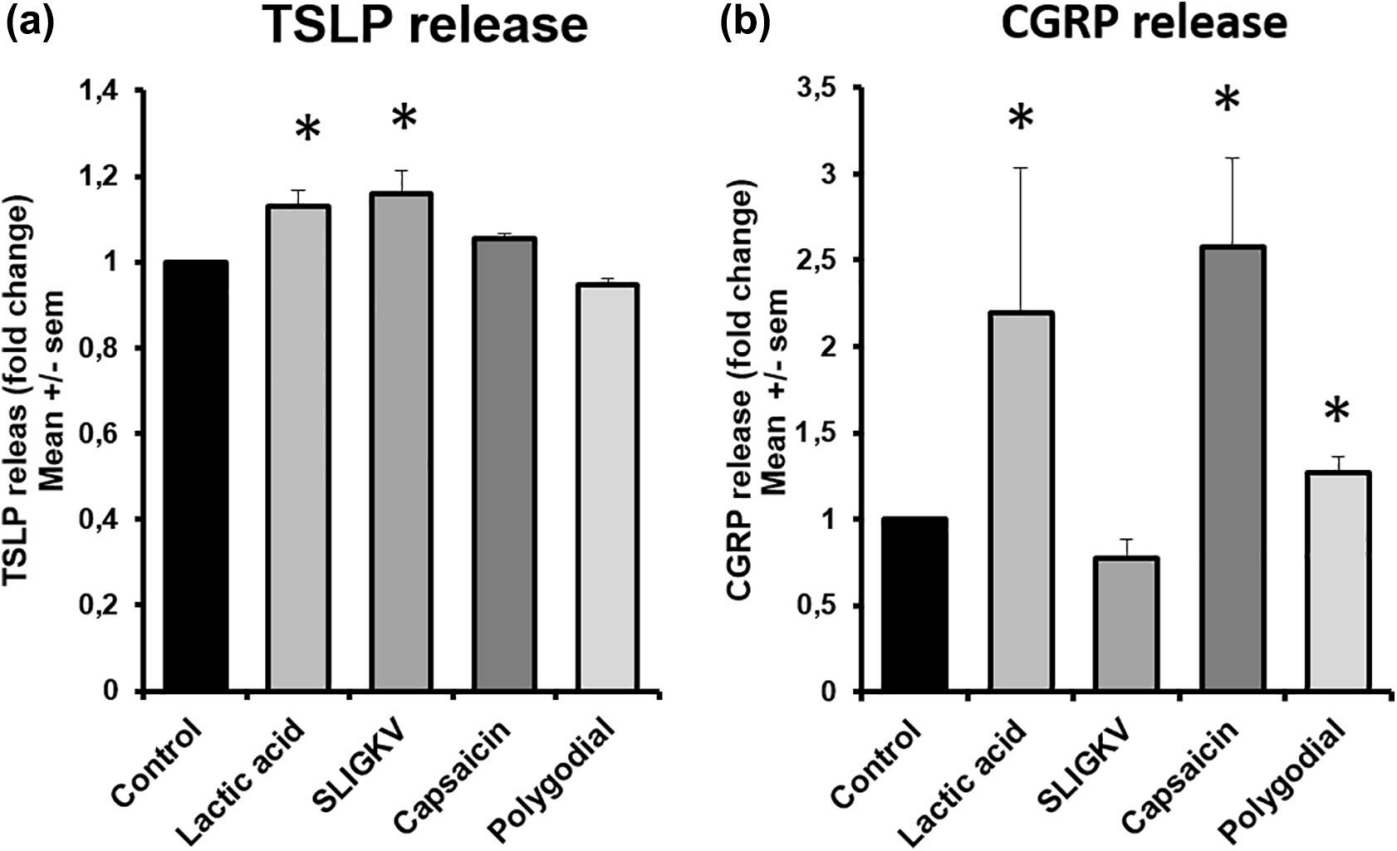
Thymic stromal lymphopoietin (TSLP) and calcitonin‐gene related peptide (CGRP) release after exposure to receptor agonists. TSLP and CGRP release 24 h after exposure of different receptor agonists in the 5 days re‐innervated skin model. Each protein was analysed with solvent as control condition. (a) TSLP analysis. (b) CGRP analysis. *p < 0.05; TSLP: *n* = 4 SLIGKV, polygodial *n* = 3 AL, Capsaicin; CGRP: *n* = 3. Values present mean ± SEM

In the last set of experiments, we dosed the TSLP and CGRP release after exposure to different receptor agonists (Figure 6). TSLP release was significantly induced by LA and SLIGKV (Figure 6a). The CGRP release was significantly induced by capsaicin, by LA and polygodial (Figure 6b).

## 3 DISCUSSION

In this model, we were able to demonstrate the presence of new nerve fibres in skin explants at day 5 of co‐culture with sensory neurons. Skin homoeostasis was impacted by the presence of neurons: epidermal apoptosis was normalized and epidermal thickness was stabilized. Presence of major actors of non‐histaminergic pruritus pathway such as IL31, IL31R, TSLP, TSLPR, PAR2 and TRPA1 was demonstrated by immunostaining in the re‐innervated skin model after 5 days of co‐culture. The topical use of three agonists (SLIGKV for PAR2, polygodial for TRPA1 and capsaicin for TRPV1) on the skin explant activated the re‐innervated nerve fibres. With qPCR, in epidermis, activation by SLIGKV showed a decrease of VEGF expression, polygodial an increase of TSLP, TNF‐α and NGF expression and capsaicin a decrease of sema3 and TNF‐α expression. Interestingly, By enzyme‐linked immunosorbent assay (ELISA), release of TSLP was slowly but significantly increased with capsaicin or SLIGKV addition but decreased with polygodial. Release of CGRP was significantly increased by capsaicin and polygodial. In neurons, activation by polygodial showed a decrease of EGF and BDNF expression, capsaicin a decrease of MT5‐MMP, BDNF and CGRP and LA a decrease of EGF.

We were able to show that epidermal apoptosis was decreased and epidermal thickness was stabilized with coculture of sensory neurons confirming the role of neuron in epidermal homoeostasis. In our previous model we demonstrated the role of sensory innervation in regulation of apoptosis.^6^ Interestingly the apoptosis was normalized in our current model. In a previous study we showed than innervation induced an increase of epidermal thickness in reinnervated conditions. However, in our optimized model we demonstrated a decrease of epidermal thickness compared to non‐innervated condition, approaching the current model to physiological conditions. The difference may be explained by a more preservative effect of neurons in current culture conditions due to an optimized and shorter re‐innervation.

In order to study inflammation and the non‐histaminic itch pathway, we analysed the expression of signals and their modulation. Regarding the epidermal PAR2/TSLP pathway, we could show that the activation of PAR2 by SLIGKV induced an increase of TSLP‐release in the supernatant. However, the modulation of transcript was not modified by activation and TSLP was already expressed in the epidermis, suggesting that TSLP is induced by the culture conditions. CGRP release was induced by TRPV1 and TRPA1 agonist. LA induced the release of TSLP and CGRP. *In vivo* LA test provokes unpleasant stinging and itch sensation.

To summarize our results, we observed that activation of TRPV1, TRPA1 and PAR‐2 led to different response‐profiles in skin explant and neurons in accordance with physiopathological conditions. The present model allows the differential study of the non‐histaminergic pruritus pathway with the use of different techniques simultaneously, so far not impossible in human or 2D models. However, currently, it is difficult to distinguish specifically itch, pain or neurogenic inflammation *in vitro* due to the common cytokine or receptor. To distinguish and study one or the other sensation, it is possible to induce pathway with molecule or receptor known to be specifically induce pain or itch (e.g. the activation of PAR2 by SLIGKV induces itch). If the molecule or receptor is not clearly known in pain or itch, the analyse of specific and no specific marker of each can help in the analysis to decipher the pathway (e.g. the dosage of TSLP release by keratinocytes for one of multiple pathways of itch).

Like all models, this one has some limitations. The main limitation is that this model is not convenient for the screening of a large number of molecules.^26^, ^35^ There is a need for careful and time‐consuming preparation and maintenance of this model and we would rather use a coculture of neurons and keratinocytes for screening. Another limitation could be that DRG cells are not only neurons but also comprise cells of their environment like glial cells. In our opinion, this is rather an advantage because neurons are less effective in co‐culture with skin in the absence of glial cells.^10^, ^36^ Moreover, different types of central and peripheral glial cells may be differentially involved in the development of chronic itch.^37^ In our model only peripheral glial cells are present, knowing this the model can be referred as re‐innervated peripheral model. Connection or integration with central glial cells like microglia and astrocytes or central neuron could be considered.

In the recent years, promising results were provided on the pathophysiology of itch and neurogenic skin inflammation.^20^, ^38^ However, most of them were obtained on animal models or based on monotypic cell cultures. This model allows the study of precise mechanisms in close‐to‐physiology conditions. Furthermore, pre‐selected active substances could be screened in this model before *in vivo* experiments.

## 4 MATERIALS AND METHODS

### 4.1 Cell culture

#### 4.1.1 Human skin explant

The donors of human skin from abdominal reduction surgery gave their written informed consent. The study was approved by the local ethics committee. The explanted skin samples were washed with ethanol 70% and subsequently with phosphate‐buffered saline (PBS). About 5‐mm punch biopsies were performed and placed in culture medium (see below) in transwell insert alone or on DRG sensory neurons at the air–liquid interface. Figure S1 shows the schematic representation of re‐innervated skin model.

Skin explants were collected at different time points of culture: baseline before incubation (D0) or after 5 or 8 days of culture/co‐culture (D5 and D8). The samples were fixed in 4% paraformaldehyde solution and cryopreserved in 10% sucrose solution. The blocks were frozen in isopentane, cooled with liquid nitrogen and conserved at −80°C. Skin explants were cryostat‐sectioned with a thickness of 30 μm in the centre of skin explant (for nerve fibres observation) or 10 μm of thickness (for other immunohistochemistry [IHC] analysis). Skin slices were preserved at −20°C until staining for a maximum of 15 days. Each culture condition was realized in duplicate.

#### 4.1.2 Dorsal root ganglia preparation

DRG cells were extracted from new‐born Wistar rats (aged 2–5 days). DRG were enzymatically dissociated for 35 min in 250 μg/ml collagenase IV solution (dissolved in DMEM) before mechanical dissection with a pipette. The equivalent of a half of all DRG cells was seeded on one transwell insert (Corning 3406) pre‐coated with 5 μg/ml. Poly‐L‐lysin (Sigma). The culture conditions for neurons alone: DMEM/F12 3/1 (50% of DMEM/F12 and 50% of DMEM) supplemented with Normocin™ 500X, NGF 100 ng/ml, insulin 4 μg/ml, hydrocortisone 10 ng/ml, B27 50X and BDNF 25 ng/ml for 2 days or 5–15 days (medium was replaced once per week). About 500 μl in transwell and 1 ml in well were used. DRG plus skin explant: after 7–15, 2 or 0 days in DMEM/F12 3/1 (50% of DMEM/F12 and 50% of DMEM) supplemented with Normocin™ 500X, NGF 25 ng/ml, insulin, hydrocortisone for 5 or 8 days. The medium was replaced every 2–3 days.

### 4.2 Histology

#### 4.2.1 Nerve fibre staining (PGP9.5 immunostaining)

After drying, incubation was performed with PBS containing 10% bovine serum albumin (BSA) for 30 min. The solution (PBS, BSA 1% with 0.3% Triton X‐100) containing the primary antibody against PGP9.5 (1/100, Ab27053, Abcam) was applied overnight at RT in a humidified chamber. Tissue was rinsed three times for 5 min in DPBS Tween 1/1000. Subsequently the sample was incubated at RT in the dark for 2 h with the secondary antibody solution coupled to a fluorochrome (Jackson Immunoresearch, 111‐095‐003 1/50) in PBS, BSA at 1%. After washing, the preparation was mounted between slide and coverslip with a hydrophilic liquid containing DAPI (Prolong® Gold antifade reagent). Tissues were observed under the fluorescence microscope. A control without primary antibody was realized.

For evaluation of nerve fibre density, the number of nerve fibres in the epidermis was counted in the entire slice and divided by the length of the epidermis to obtain a number of fibre per mm. The size of slices was of 30 μm, to obtain a density of nerve fibres per millimetre square this number was multiplied by 1/0.030 (1 mm/30 μm). The number of fibres was evaluated for three slices of skin explant (two by condition), and a mean was calculated.

#### 4.2.2 Immunohistology

After drying, saturation and permeabilization was performed by incubation for 15 min with a solution of PBS containing 5% of normal donkey serum, 0.1% of saponin and 0.05% of Triton X‐100. The solution (PBS, 2% donkey serum, 0.1% saponin) with primary antibody against the specific antigens (Table S1) was applied overnight at 4°C (except for PAR2 at RT) in humidified chambers. Then, the cells were rinsed three times for 5 min in DPBS Tween 1/1000 and incubated at RT in the dark for 2 h with the secondary antibody solution coupled to a fluorochrome (Table S1) in PBS, BSA at 1%. After washing, the preparation was mounted between slide and coverslip with a hydrophilic liquid containing DAPI (Prolong® Gold antifade reagent). Cells were observed under the fluorescence microscope. In all manipulations a control without primary antibody was realized. Isotype control was checked.

#### 4.2.3 Haematoxylin‐eosin‐safran (HES) staining

Slices of skin were stained with classic HES Stain. For evaluation of epidermal thickness, a picture of the epidermis by duplicate for each condition was taken at the centre of the skin explant using. The total volume seen of epidermis of the skin explant was divided by the total length of epidermis of skin explant to obtain the average thickness of epidermis.

#### 4.2.4 Apoptosis evaluation (TUNEL)

After drying, the slices of skin were permeabilized for 2 min at 4°C using PBS solution with 0.1% Triton X‐100 and 0.1% sodium citrate. Slices were washed two times with PBS. About 50 μl of TUNEL reaction mixture (*In Situ* Cell Death Detection Kit, Fluorescein, Roche) was applied for 1 h at 37°C in the dark. Tissues were washed three times with PBST and mounted with DAPI. Control without apoptosis (D0) was used. For each condition (and in duplicate), three random fields of skin epidermis on different slices were pictured with a 20× objective with a filter for DAPI and a filter for Fluorescein (apoptotic cells). On each picture total number of cells (DAPI, around 400 cells) and total number of apoptotic cells (green) were counted. Percentage of apoptotic cells were calculated.

### 4.3 qPCR

mRNA from skin explant or DRG from the model were obtained using Tri‐reagent. The OD at 280/260 and 260/230 ratio were checked. RT‐qPCR experiments were realized using a standard kit (Applied Biosystems™, High capacity cDNA Reverse Transcriptase Kit). QPCR experiments were realized using Power SYBR® Green PCR Master Mix on StepOne™ qPCR. qPCR probes were in‐house homemade designed or taken from public libraries (Table S2). For validation of probe and specific amplification melt curves were realized. A control without cDNA was performed (only the mix and probe). The result was presented in Ct or in fold change using actin as housekeeping gene.

qPCR was realized on skin explant and DRG cells of the re‐innervated skin model. In parallel, the DRG present in the bottom of the culture was dissected and treated for qPCR. To avoid potential contamination between skin and neuronal cell, specific probes of human and rats were used.

### 4.4 Patch clamp

Ionic currents were recorded in nerve fibres attached in a cell‐attached configuration by using a macro‐patch clamp technique at RT (22 ± 2°C). The approach of the pipette (Clark Electromedical Glass) 1.5 mm in diameter, 3 µm at the tip, was monitored with an inverted microscope (Olympus IX 70) equipped with Hoffman contrast and a progressive‐scan digital camera (XC8500CE, Sony, Kanagawa, Japan). Patch‐clamp experiments were performed in a fresh culture medium: DMEM/F12 3/1 (50% of DMEM/F12 and 50% of DMEM) supplemented with Normocin™ 500X, NGF 25 ng/ml, insulin, hydrocortisone. About 5µl of capsaicin (at 10 μM) was used for the activation of the TRPV1, SLIGKV (at 100 μM) used for the activation of the PAR2, polygodial for TRPA1 (3 μM) and LA (10%). Solutions with agonists were topically applied at the surface of the epidermis of re‐innervated skin explants as previously published.^39^ Spontaneous current was recorded, on fibre attached at the skin explant and selected randomly, all along the manipulation, several minutes before the application of agonists until several minutes after the application of agonists. The number of spikes was evaluated under the same duration after and before the topic application for each recording. A peak was considered with a threshold of 0.1 nA.

### 4.5 ELISA

ELISA were realized according the manufacturer's instructions. For TSLP, the DuoSet by R&D was used (DY1398‐05). For CGRP, the kit from Interchim was used (Interchim, A05481, Montluçon, France).

### 4.6 Statistics

Since the present series of studies are of exploratory character no form statistical analysis plan was elaborated. In case of low numbers in the sample Mann–Whitney *U*‐test for unpaired samples. In case of a sufficient large sample after testing for normality (Kolmogorov–Smirnov test) a *t*‐test was selected. To validate the model, and due to the previously published known of positive effect of neuron on the skin homoeostasis and the role of agonist on production of proinflammatory mediators, one‐tailed tests were realized. Due to the exploratory nature of the study no correction for an alpha‐error was performed. Prism 5 (GraphPad) was used as statistic program. *n* represent independent experiment.

## CONFLICT OF INTEREST

N. Lebonvallet has received research grant from J & J. C. Brun and T. Oddos are employees of J & J. L. Misery was a consultant for Beiersdorf, Bioderma, Clarins, Expanscience, Johnson & Johnson, L'Oréal, Nestlé Skin Health, Pierre Fabre, Solabia and Uriage. J. W. Fluhr was a consultant or speaker for Pierre Fabre, Sebapharma, Nestlé Skin Health, Bioderma and Expanscience.

## AUTHOR CONTRIBUTIONS

**N. Lebonvallet:** Conceptualization; Investigation; Methodology; Project administration; Validation; Writing – original draft; Writing – review & editing. **J. W. Fluhr:** Validation; Writing – original draft; Writing – review & editing. **C. Le Gall‐Ianotto:** Investigation; Methodology; Project administration. **R. Leschiera:** Formal analysis; Methodology; Project administration. **M. Talagas:** Methodology; Project administration. **A. Reux:** Formal analysis; Investigation; Methodology. **A. Bataille:** Methodology. **C. Brun:** Funding acquisition; Investigation; Validation; Writing – review & editing. **T. Oddos:** Funding acquisition; Validation; Writing – review & editing. **J.‐P. Pennec:** Formal analysis; Investigation; Methodology. **J.‐L. Carré:** Project administration; Supervision; Validation. **L. Misery:** Conceptualization; Project administration; Supervision; Validation; Writing – review & editing.

## Supporting information

Supporting Information S1

## Data Availability

Data available on request from the authors.
