# Supplementary material for: A re‐innervated in vitro skin model of non‐histaminergic itch and skin neurogenic inflammation: PAR2‐, TRPV1‐ and TRPA1‐agonist induced functionality
Source: Skin Health Dis. 2021 Sep 30;1(4):e66. doi: 10.1002/ski2.66 (PMC9060135; doi:10.1002/ski2.66)
Supplement: Supplementary file 1 — Supporting Information S1 [file SKI2-1-e66-s001.docx]

Supplementary Materials

Materials and Methods


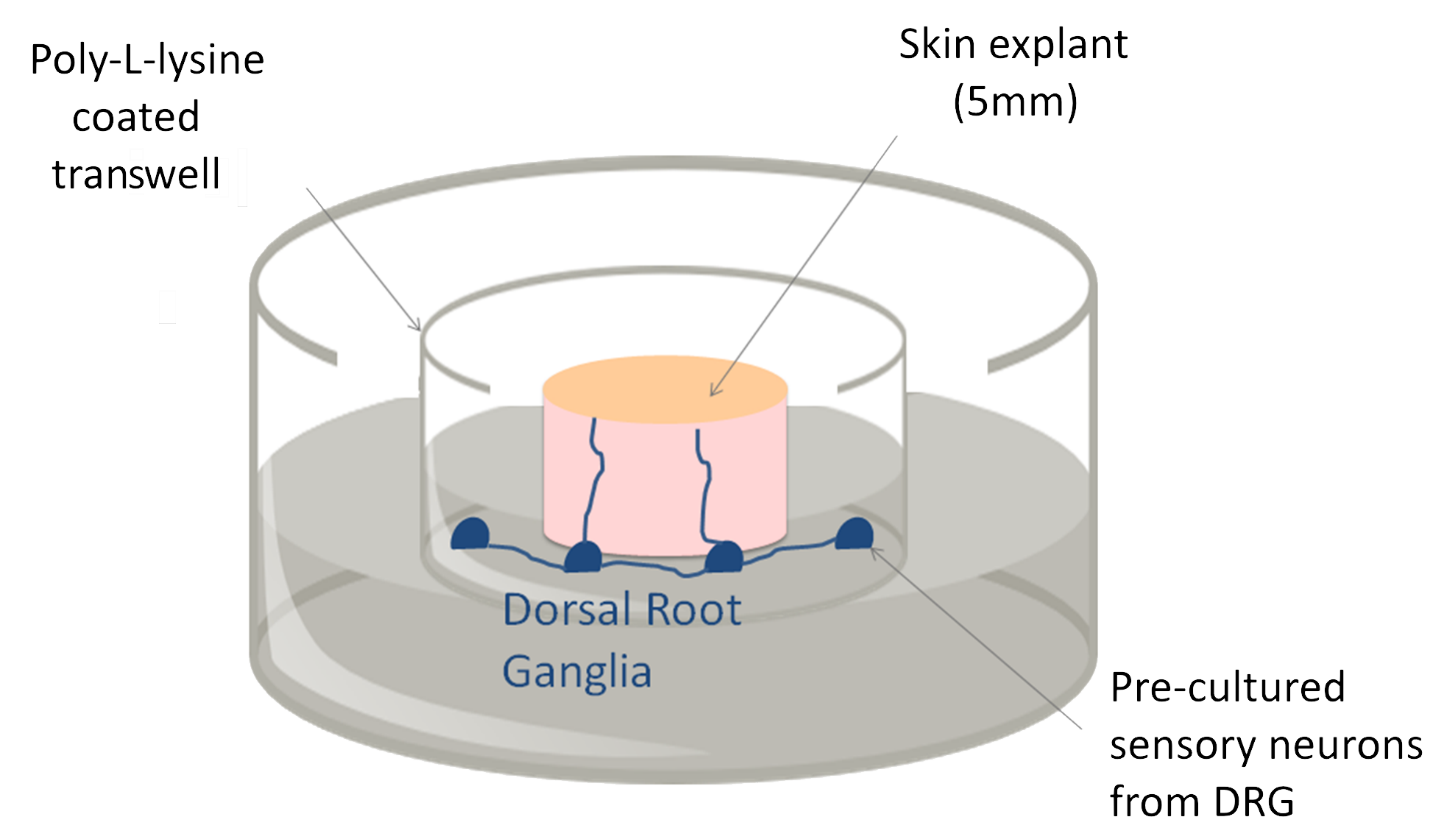


**Supplementary Fig. 1: Schematic representation of re-innervated 3D skin model.**

Isolated sensory neurons from rodent DRG were seeded in a poly-L-Lysin coated transwell and maintained in culture for a minimum of five days. Subsequently, human skin explant was placed for re-innervation on the network of neurons up to 8 days.

**Supplementary Table 1:** List of antibodies used for immunohistochemistry

| **Antibody** | **Isotype, species, dilution, incubation** | **Reference** |
| --- | --- | --- |
| **Primary antibody :** |  |  |
| - **PAR2** - **TSLP** | IgG, Polyclonal, Rabbit, 1/50, RT  IgG, Polyclonal, Rabbit, 1/100 | Abcam, 180953  Abcam, 47943 |
| - **IL31R** | IgG, Polyclonal, Rabbit, 1/200 | Santacruz, 135154 |
| - **TRPA1** | IgG, Polyclonal, Rabbit, 1/100 | Abcam, 58844 |
| - **TSLPR** | IgG, Polyclonal, Rabbit, 1/100 | Abcam, ab109626 |
| - **PGP9.5** | IgG, Polyclonal, Rabbit, 1/100 | Abcam, ab27053 |
| - **PGP9.5** | IgG, Monoclonal, Mouse, 1/50 | Santacruz, 20559 |
| - **Isotypic control** | IgG, Polyclonal, Rabbit | Abcam 27478 |
| **Secondary antibody :** |  |  |
| - **Anti-Rabbit FITC** | IgG, Goat, 1/50 | 111-095-003, Jackson |
| - **Anti-Rabbit Chromeo 488** | IgG, Goat, 1/300 | Abcam, a60314 |
| - **Anti-Mouse TRITC** | IgG, Goat, 1/300 | Sigma, T7782 |

**Supplementary Table 2:** List of probes used for qPCR analysis on neuron (rat) or skin explant (human)

|  | Probe (Forward/Reverse)  5’ -> 3’ | Hybridation/Elongation |
| --- | --- | --- |
| Human NGF | ATA-CAG-GCG-GAA-CCA-CAC-TC  TGC-TCC-TGT-GAG-TCC-TGT-TG | 60°C |
| Human Sema3A | ACC-ACC-CAA-TCA-GGA-CAG-AG  TGG-CAC-TGA-GCA-AAT-CAG-AC | 60°C |
| Human TSLP | CCA-GAA-AGC-TCT-GGA-GCA-TCA  ACC-CAA-TTC-CAC-CCC-AGT-TT | 60°C |
| Human TNFa | AGA-ACT-CAC-TGG-GGC-CTA-CA  GCT-CCG-TGT-CTC-AAG-GAA-GT | 60°C |
| Human VEGF | CTA CCT CCA CCA TGC CAA GT  TGG TGA TGT TGG ACT CCT CA | 60°C |
| Rat BDNF | GGC-CCA-ACG-AAG-AAA-AC  AGC-ATC-ACC-CGG-GAA-GTG-T | 60°C |
| Rat CALCA | CCT-TTC-CTG-GTT-GTC-AGC-ATC-TT  CAG-TAG-GCG-AGC-TTC-TTC-TTC-AC | 50°C |
| Rat EGF | CCC-GTG-TTC-TTC-TGA-GTT-CC  TGT-AAC-CGT-GGC-TTC-CTT-CT | 60°C |
| Rat MT5-MMP | TTC-CCC-AGG-CAC-TAA-GGA-CT  GTC-ACA-AAC-TTG-GAT-CCG-CC | 60°C |
| Rat TNFa | CAT-CCG-TTC-TCT-ACC-CAG-CC  CCC-AGA-GCC-ACA-ATT-CCC-TT | 60°C |
| Actin | GAG-ACC-TTC-AAC-ACC-CCA-GC  ATG-TCA-CGC-ACG-ATT-TCC-CT | 60°C |
